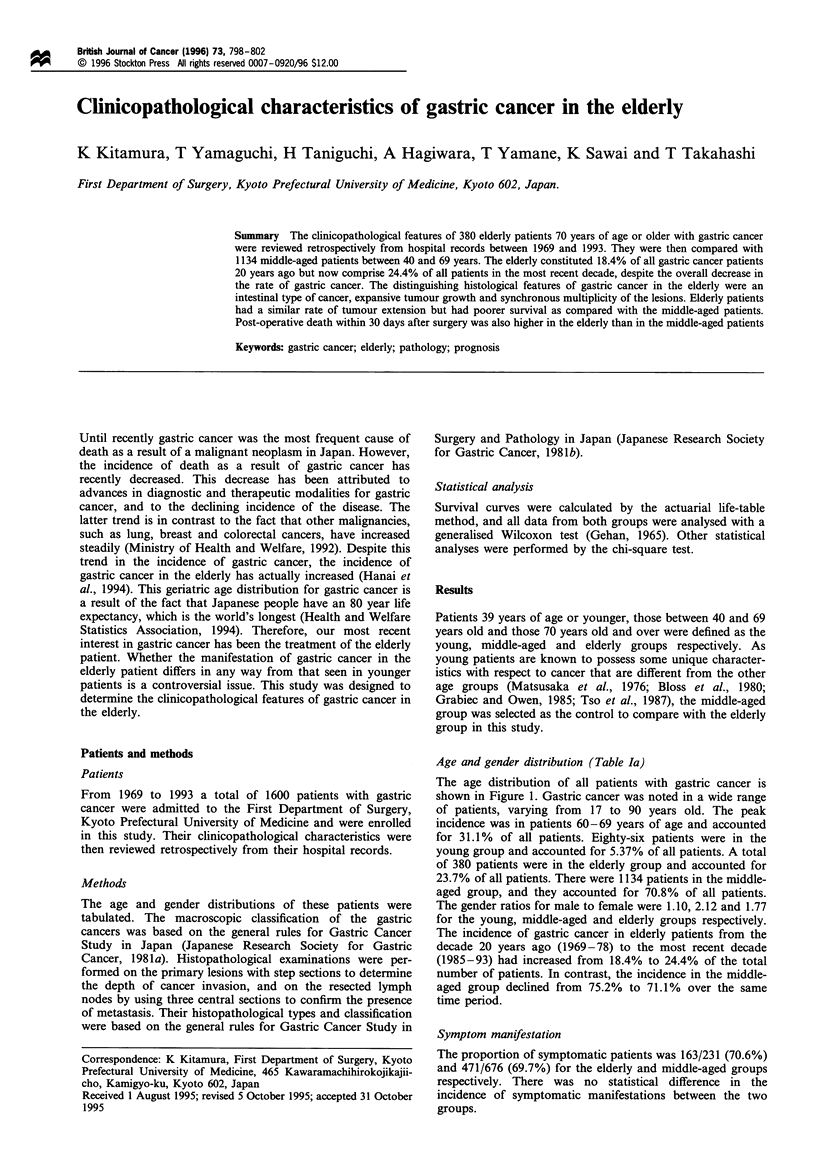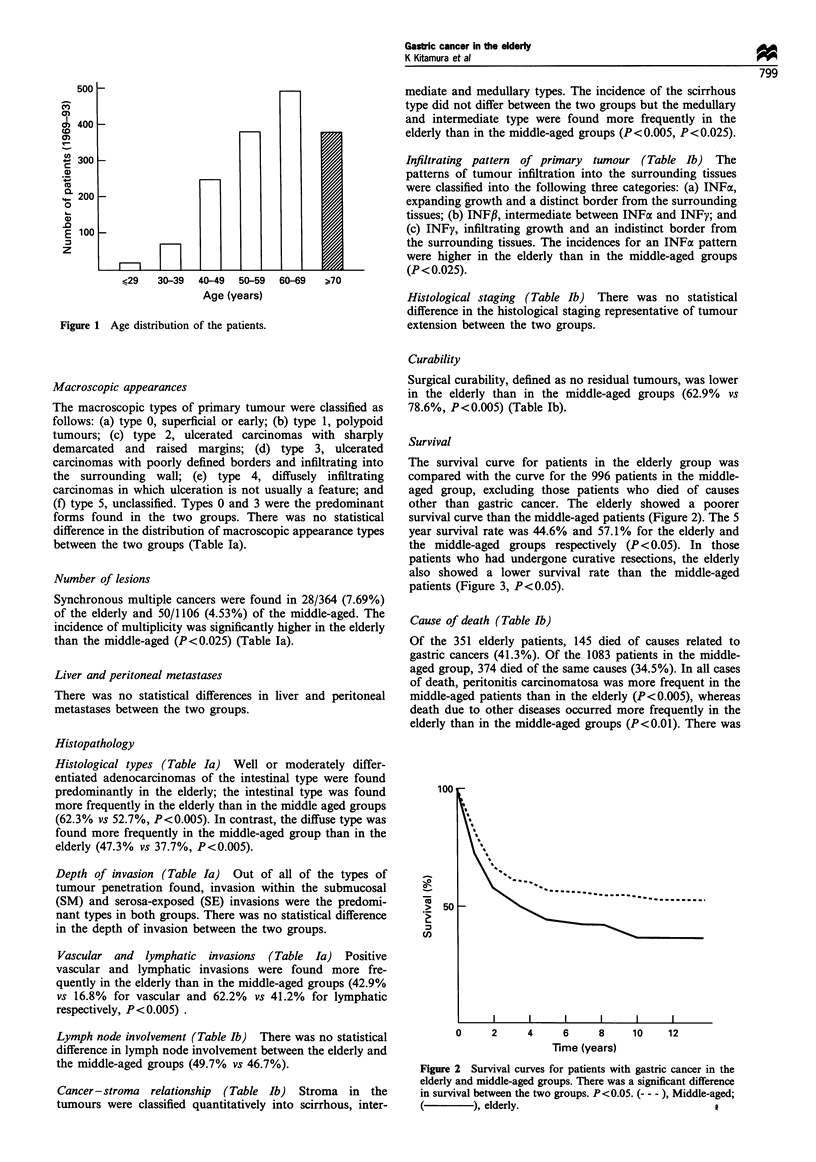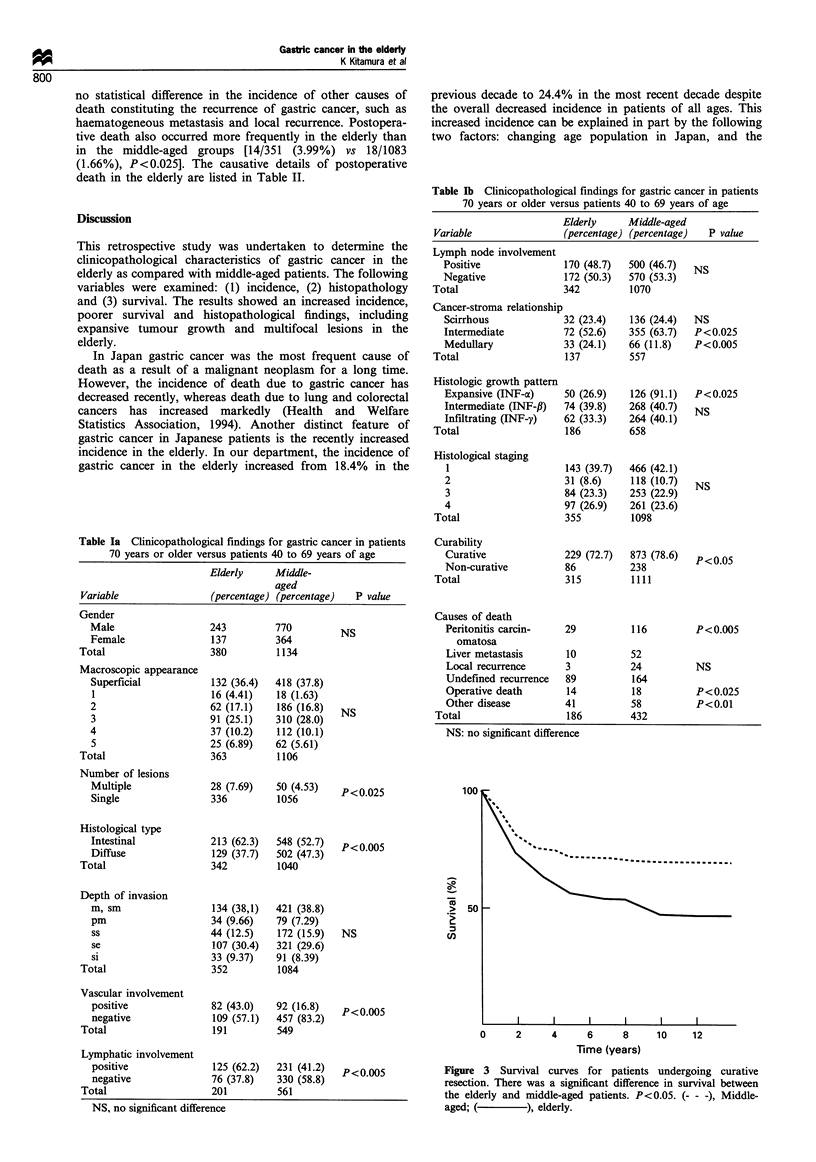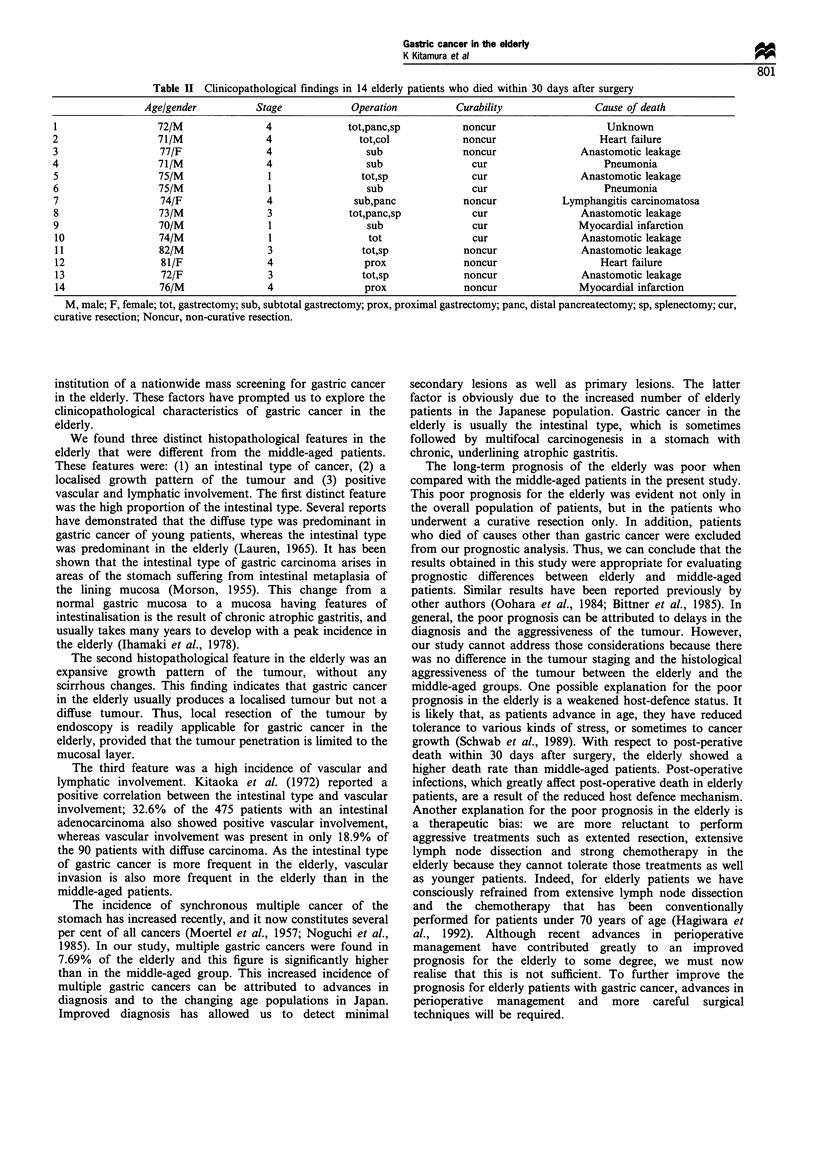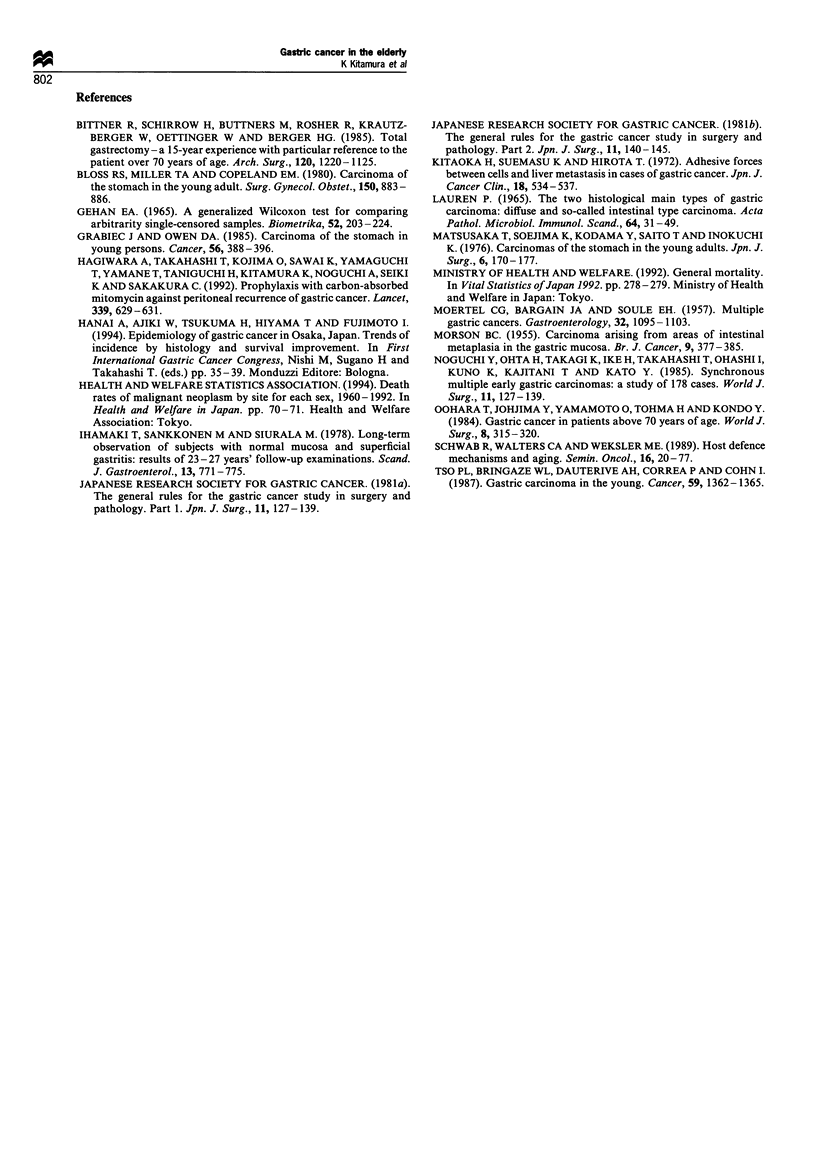# Clinicopathological characteristics of gastric cancer in the elderly.

**DOI:** 10.1038/bjc.1996.139

**Published:** 1996-03

**Authors:** K. Kitamura, T. Yamaguchi, H. Taniguchi, A. Hagiwara, T. Yamane, K. Sawai, T. Takahashi

**Affiliations:** First Department of Surgery, Kyoto Prefectural University of Medicine, Japan.

## Abstract

The clinicopathological features of 380 elderly patients 70 years of age or older with gastric cancer were reviewed retrospectively from hospital records between 1969 and 1993. They were then compared with 1134 middle-aged patients between 40 and 69 years. The elderly constituted 18.4% of all gastric cancer patients 20 years ago but now comprise 24.4% of all patients in the most recent decade, despite the overall decrease in the rate of gastric cancer. The distinguishing histological features of gastric cancer in the elderly were an intestinal type of cancer, expansive tumour growth and synchronous multiplicity of the lesions. Elderly patients had a similar rate of tumour extension but had poorer survival as compared with the middle-aged patients. Post-operative death within 30 days after surgery was also higher in the elderly than in the middle-aged patients.


British Journal of Cancer (1996) 73, 798-802
© 1996 Stockton Press All rights reserved 0007-0920/96 $12.00

### Clinicopathological characteristics of gastric cancer in the elderly

K Kitamura, T Yamaguchi, H Taniguchi, A Hagiwara, T Yamane, K Sawai and T Takahashi

*First Department of Surgery, Kyoto Prefectural University of Medicine, Kyoto 602, Japan.*

**Summary** The clinicopathological features of 380 elderly patients 70 years of age or older with gastric cancer were reviewed retrospectively from hospital records between 1969 and 1993. They were then compared with 1134 middle-aged patients between 40 and 69 years. The elderly constituted 18.4% of all gastric cancer patients 20 years ago but now comprise 24.4% of all patients in the most recent decade, despite the overall decrease in the rate of gastric cancer. The distinguishing histological features of gastric cancer in the elderly were an intestinal type of cancer, expansive tumour growth and synchronous multiplicity of the lesions. Elderly patients had a similar rate of tumour extension but had poorer survival as compared with the middle-aged patients. Post-operative death within 30 days after surgery was also higher in the elderly than in the middle-aged patients

**Keywords:** gastric cancer; elderly; pathology; prognosis

Until recently gastric cancer was the most frequent cause of death as a result of a malignant neoplasm in Japan. However, the incidence of death as a result of gastric cancer has recently decreased. This decrease has been attributed to advances in diagnostic and therapeutic modalities for gastric cancer, and to the declining incidence of the disease. The latter trend is in contrast to the fact that other malignancies, such as lung, breast and colorectal cancers, have increased steadily (Ministry of Health and Welfare, 1992). Despite this trend in the incidence of gastric cancer, the incidence of gastric cancer in the elderly has actually increased (Hanai *et al.*, 1994). This geriatric age distribution for gastric cancer is a result of the fact that Japanese people have an 80 year life expectancy, which is the world's longest (Health and Welfare Statistics Association, 1994). Therefore, our most recent interest in gastric cancer has been the treatment of the elderly patient. Whether the manifestation of gastric cancer in the elderly patient differs in any way from that seen in younger patients is a controversial issue. This study was designed to determine the clinicopathological features of gastric cancer in the elderly.

### Patients and methods

### Patients

From 1969 to 1993 a total of 1600 patients with gastric cancer were admitted to the First Department of Surgery, Kyoto Prefectural University of Medicine and were enrolled in this study. Their clinicopathological characteristics were then reviewed retrospectively from their hospital records.

### Methods

The age and gender distributions of these patients were tabulated. The macroscopic classification of the gastric cancers was based on the general rules for Gastric Cancer Study in Japan (Japanese Research Society for Gastric Cancer, 1981*a*). Histopathological examinations were performed on the primary lesions with step sections to determine the depth of cancer invasion, and on the resected lymph nodes by using three central sections to confirm the presence of metastasis. Their histopathological types and classification were based on the general rules for Gastric Cancer Study in Surgery and Pathology in Japan (Japanese Research Society for Gastric Cancer, 1981*b*).

### Statistical analysis

Survival curves were calculated by the actuarial life-table method, and all data from both groups were analysed with a generalised Wilcoxon test (Gehan, 1965). Other statistical analyses were performed by the chi-square test.

### Results

Patients 39 years of age or younger, those between 40 and 69 years old and those 70 years old and over were defined as the young, middle-aged and elderly groups respectively. As young patients are known to possess some unique characteristics with respect to cancer that are different from the other age groups (Matsusaka *et al.*, 1976; Bloss *et al.*, 1980; Grabiec and Owen, 1985; Tso *et al.*, 1987), the middle-aged group was selected as the control to compare with the elderly group in this study.

### Age and gender distribution (Table Ia)

The age distribution of all patients with gastric cancer is shown in Figure 1. Gastric cancer was noted in a wide range of patients, varying from 17 to 90 years old. The peak incidence was in patients 60-69 years of age and accounted for 31.1% of all patients. Eighty-six patients were in the young group and accounted for 5.37% of all patients. A total of 380 patients were in the elderly group and accounted for 23.7% of all patients. There were 1134 patients in the middle-aged group, and they accounted for 70.8% of all patients. The gender ratios for male to female were 1.10, 2.12 and 1.77 for the young, middle-aged and elderly groups respectively. The incidence of gastric cancer in elderly patients from the decade 20 years ago (1969-78) to the most recent decade (1985-93) had increased from 18.4% to 24.4% of the total number of patients. In contrast, the incidence in the middle-aged group declined from 75.2% to 71.1% over the same time period.

### Symptom manifestation

The proportion of symptomatic patients was 163/231 (70.6%) and 471/676 (69.7%) for the elderly and middle-aged groups respectively. There was no statistical difference in the incidence of symptomatic manifestations between the two groups.

Correspondence: K Kitamura, First Department of Surgery, Kyoto Prefectural University of Medicine, 465 Kawaramachihirokojikajii-cho, Kamigyo-ku, Kyoto 602, Japan
Received 1 August 1995; revised 5 October 1995; accepted 31 October 1995

**Figure 1** Age distribution of the patients.

*Macroscopic appearances*

The macroscopic types of primary tumour were classified as follows: (a) type 0, superficial or early; (b) type 1, polypoid tumours; (c) type 2, ulcerated carcinomas with sharply demarcated and raised margins; (d) type 3, ulcerated carcinomas with poorly defined borders and infiltrating into the surrounding wall; (e) type 4, diffusely infiltrating carcinomas in which ulceration is not usually a feature; and (f) type 5, unclassified. Types 0 and 3 were the predominant forms found in the two groups. There was no statistical difference in the distribution of macroscopic appearance types between the two groups (Table Ia).

*Number of lesions*

Synchronous multiple cancers were found in 28/364 (7.69%) of the elderly and 50/1106 (4.53%) of the middle-aged. The incidence of multiplicity was significantly higher in the elderly than the middle-aged ($P<0.025$) (Table Ia).

*Liver and peritoneal metastases*

There was no statistical differences in liver and peritoneal metastases between the two groups.

*Histopathology*

*Histological types (Table Ia)* Well or moderately differentiated adenocarcinomas of the intestinal type were found predominantly in the elderly; the intestinal type was found more frequently in the elderly than in the middle aged groups (62.3% *vs* 52.7%, $P<0.005$). In contrast, the diffuse type was found more frequently in the middle-aged group than in the elderly (47.3% *vs* 37.7%, $P<0.005$).

*Depth of invasion (Table Ia)* Out of all of the types of tumour penetration found, invasion within the submucosal (SM) and serosa-exposed (SE) invasions were the predominant types in both groups. There was no statistical difference in the depth of invasion between the two groups.

*Vascular and lymphatic invasions (Table Ia)* Positive vascular and lymphatic invasions were found more frequently in the elderly than in the middle-aged groups (42.9% *vs* 16.8% for vascular and 62.2% *vs* 41.2% for lymphatic respectively, $P<0.005$) .

*Lymph node involvement (Table Ib)* There was no statistical difference in lymph node involvement between the elderly and the middle-aged groups (49.7% *vs* 46.7%).

*Cancer–stroma relationship (Table Ib)* Stroma in the tumours were classified quantitatively into scirrhous, intermediate and medullary types. The incidence of the scirrhous type did not differ between the two groups but the medullary and intermediate type were found more frequently in the elderly than in the middle-aged groups ($P<0.005$, $P<0.025$).

*Infiltrating pattern of primary tumour (Table Ib)* The patterns of tumour infiltration into the surrounding tissues were classified into the following three categories: (a) INFα, expanding growth and a distinct border from the surrounding tissues; (b) INFβ, intermediate between INFα and INFγ; and (c) INFγ, infiltrating growth and an indistinct border from the surrounding tissues. The incidences for an INFα pattern were higher in the elderly than in the middle-aged groups ($P<0.025$).

*Histological staging (Table Ib)* There was no statistical difference in the histological staging representative of tumour extension between the two groups.

*Curability*

Surgical curability, defined as no residual tumours, was lower in the elderly than in the middle-aged groups (62.9% *vs* 78.6%, $P<0.005$) (Table Ib).

*Survival*

The survival curve for patients in the elderly group was compared with the curve for the 996 patients in the middle-aged group, excluding those patients who died of causes other than gastric cancer. The elderly showed a poorer survival curve than the middle-aged patients (Figure 2). The 5 year survival rate was 44.6% and 57.1% for the elderly and the middle-aged groups respectively ($P<0.05$). In those patients who had undergone curative resections, the elderly also showed a lower survival rate than the middle-aged patients (Figure 3, $P<0.05$).

*Cause of death (Table Ib)*

Of the 351 elderly patients, 145 died of causes related to gastric cancers (41.3%). Of the 1083 patients in the middle-aged group, 374 died of the same causes (34.5%). In all cases of death, peritonitis carcinomatosa was more frequent in the middle-aged patients than in the elderly ($P<0.005$), whereas death due to other diseases occurred more frequently in the elderly than in the middle-aged groups ($P<0.01$). There was

**Figure 2** Survival curves for patients with gastric cancer in the elderly and middle-aged groups. There was a significant difference in survival between the two groups. $P<0.05$. (- - - ), Middle-aged; (————), elderly.

no statistical difference in the incidence of other causes of death constituting the recurrence of gastric cancer, such as haematogeneous metastasis and local recurrence. Postoperative death also occurred more frequently in the elderly than in the middle-aged groups [14/351 (3.99%) *vs* 18/1083 (1.66%), $P<0.025$]. The causative details of postoperative death in the elderly are listed in Table II.

### Discussion

This retrospective study was undertaken to determine the clinicopathological characteristics of gastric cancer in the elderly as compared with middle-aged patients. The following variables were examined: (1) incidence, (2) histopathology and (3) survival. The results showed an increased incidence, poorer survival and histopathological findings, including expansive tumour growth and multifocal lesions in the elderly.

In Japan gastric cancer was the most frequent cause of death as a result of a malignant neoplasm for a long time. However, the incidence of death due to gastric cancer has decreased recently, whereas death due to lung and colorectal cancers has increased markedly (Health and Welfare Statistics Association, 1994). Another distinct feature of gastric cancer in Japanese patients is the recently increased incidence in the elderly. In our department, the incidence of gastric cancer in the elderly increased from 18.4% in the previous decade to 24.4% in the most recent decade despite the overall decreased incidence in patients of all ages. This increased incidence can be explained in part by the following two factors: changing age population in Japan, and the

**Table Ia** Clinicopathological findings for gastric cancer in patients 70 years or older versus patients 40 to 69 years of age

| *Variable* | *Elderly (percentage)* | *Middle-aged (percentage)* | P *value* |
|---|---|---|---|
| Gender | | | |
| Male | 243 | 770 | NS |
| Female | 137 | 364 | |
| Total | 380 | 1134 | |
| Macroscopic appearance | | | |
| Superficial | 132 (36.4) | 418 (37.8) | |
| 1 | 16 (4.41) | 18 (1.63) | |
| 2 | 62 (17.1) | 186 (16.8) | NS |
| 3 | 91 (25.1) | 310 (28.0) | |
| 4 | 37 (10.2) | 112 (10.1) | |
| 5 | 25 (6.89) | 62 (5.61) | |
| Total | 363 | 1106 | |
| Number of lesions | | | |
| Multiple | 28 (7.69) | 50 (4.53) | $P<0.025$ |
| Single | 336 | 1056 | |
| Histological type | | | |
| Intestinal | 213 (62.3) | 548 (52.7) | $P<0.005$ |
| Diffuse | 129 (37.7) | 502 (47.3) | |
| Total | 342 | 1040 | |
| Depth of invasion | | | |
| m, sm | 134 (38,1) | 421 (38.8) | |
| pm | 34 (9.66) | 79 (7.29) | |
| ss | 44 (12.5) | 172 (15.9) | NS |
| se | 107 (30.4) | 321 (29.6) | |
| si | 33 (9.37) | 91 (8.39) | |
| Total | 352 | 1084 | |
| Vascular involvement | | | |
| positive | 82 (43.0) | 92 (16.8) | $P<0.005$ |
| negative | 109 (57.1) | 457 (83.2) | |
| Total | 191 | 549 | |
| Lymphatic involvement | | | |
| positive | 125 (62.2) | 231 (41.2) | $P<0.005$ |
| negative | 76 (37.8) | 330 (58.8) | |
| Total | 201 | 561 | |

NS, no significant difference

**Table Ib** Clinicopathological findings for gastric cancer in patients 70 years or older versus patients 40 to 69 years of age

| *Variable* | *Elderly (percentage)* | *Middle-aged (percentage)* | P *value* |
|---|---|---|---|
| Lymph node involvement | | | |
| Positive | 170 (48.7) | 500 (46.7) | NS |
| Negative | 172 (50.3) | 570 (53.3) | |
| Total | 342 | 1070 | |
| Cancer-stroma relationship | | | |
| Scirrhous | 32 (23.4) | 136 (24.4) | NS |
| Intermediate | 72 (52.6) | 355 (63.7) | $P<0.025$ |
| Medullary | 33 (24.1) | 66 (11.8) | $P<0.005$ |
| Total | 137 | 557 | |
| Histologic growth pattern | | | |
| Expansive (INF-$\alpha$) | 50 (26.9) | 126 (91.1) | $P<0.025$ |
| Intermediate (INF-$\beta$) | 74 (39.8) | 268 (40.7) | NS |
| Infiltrating (INF-$\gamma$) | 62 (33.3) | 264 (40.1) | |
| Total | 186 | 658 | |
| Histological staging | | | |
| 1 | 143 (39.7) | 466 (42.1) | |
| 2 | 31 (8.6) | 118 (10.7) | NS |
| 3 | 84 (23.3) | 253 (22.9) | |
| 4 | 97 (26.9) | 261 (23.6) | |
| Total | 355 | 1098 | |
| Curability | | | |
| Curative | 229 (72.7) | 873 (78.6) | $P<0.05$ |
| Non-curative | 86 | 238 | |
| Total | 315 | 1111 | |
| Causes of death | | | |
| Peritonitis carcinomatosa | 29 | 116 | $P<0.005$ |
| Liver metastasis | 10 | 52 | |
| Local recurrence | 3 | 24 | NS |
| Undefined recurrence | 89 | 164 | |
| Operative death | 14 | 18 | $P<0.025$ |
| Other disease | 41 | 58 | $P<0.01$ |
| Total | 186 | 432 | |

NS: no significant difference

**Figure 3** Survival curves for patients undergoing curative resection. There was a significant difference in survival between the elderly and middle-aged patients. $P<0.05$. (- - -), Middle-aged; (————), elderly.

**Table II** Clinicopathological findings in 14 elderly patients who died within 30 days after surgery

| | *Age/gender* | *Stage* | *Operation* | *Curability* | *Cause of death* |
|---|---|---|---|---|---|
| 1 | 72/M | 4 | tot,panc,sp | noncur | Unknown |
| 2 | 71/M | 4 | tot,col | noncur | Heart failure |
| 3 | 77/F | 4 | sub | noncur | Anastomotic leakage |
| 4 | 71/M | 4 | sub | cur | Pneumonia |
| 5 | 75/M | 1 | tot,sp | cur | Anastomotic leakage |
| 6 | 75/M | 1 | sub | cur | Pneumonia |
| 7 | 74/F | 4 | sub,panc | noncur | Lymphangitis carcinomatosa |
| 8 | 73/M | 3 | tot,panc,sp | cur | Anastomotic leakage |
| 9 | 70/M | 1 | sub | cur | Myocardial infarction |
| 10 | 74/M | 1 | tot | cur | Anastomotic leakage |
| 11 | 82/M | 3 | tot,sp | noncur | Anastomotic leakage |
| 12 | 81/F | 4 | prox | noncur | Heart failure |
| 13 | 72/F | 3 | tot,sp | noncur | Anastomotic leakage |
| 14 | 76/M | 4 | prox | noncur | Myocardial infarction |

M, male; F, female; tot, gastrectomy; sub, subtotal gastrectomy; prox, proximal gastrectomy; panc, distal pancreatectomy; sp, splenectomy; cur, curative resection; Noncur, non-curative resection.

institution of a nationwide mass screening for gastric cancer in the elderly. These factors have prompted us to explore the clinicopathological characteristics of gastric cancer in the elderly.

We found three distinct histopathological features in the elderly that were different from the middle-aged patients. These features were: (1) an intestinal type of cancer, (2) a localised growth pattern of the tumour and (3) positive vascular and lymphatic involvement. The first distinct feature was the high proportion of the intestinal type. Several reports have demonstrated that the diffuse type was predominant in gastric cancer of young patients, whereas the intestinal type was predominant in the elderly (Lauren, 1965). It has been shown that the intestinal type of gastric carcinoma arises in areas of the stomach suffering from intestinal metaplasia of the lining mucosa (Morson, 1955). This change from a normal gastric mucosa to a mucosa having features of intestinalisation is the result of chronic atrophic gastritis, and usually takes many years to develop with a peak incidence in the elderly (Ihamaki *et al.*, 1978).

The second histopathological feature in the elderly was an expansive growth pattern of the tumour, without any scirrhous changes. This finding indicates that gastric cancer in the elderly usually produces a localised tumour but not a diffuse tumour. Thus, local resection of the tumour by endoscopy is readily applicable for gastric cancer in the elderly, provided that the tumour penetration is limited to the mucosal layer.

The third feature was a high incidence of vascular and lymphatic involvement. Kitaoka *et al.* (1972) reported a positive correlation between the intestinal type and vascular involvement; 32.6% of the 475 patients with an intestinal adenocarcinoma also showed positive vascular involvement, whereas vascular involvement was present in only 18.9% of the 90 patients with diffuse carcinoma. As the intestinal type of gastric cancer is more frequent in the elderly, vascular invasion is also more frequent in the elderly than in the middle-aged patients.

The incidence of synchronous multiple cancer of the stomach has increased recently, and it now constitutes several per cent of all cancers (Moertel *et al.*, 1957; Noguchi *et al.*, 1985). In our study, multiple gastric cancers were found in 7.69% of the elderly and this figure is significantly higher than in the middle-aged group. This increased incidence of multiple gastric cancers can be attributed to advances in diagnosis and to the changing age populations in Japan. Improved diagnosis has allowed us to detect minimal secondary lesions as well as primary lesions. The latter factor is obviously due to the increased number of elderly patients in the Japanese population. Gastric cancer in the elderly is usually the intestinal type, which is sometimes followed by multifocal carcinogenesis in a stomach with chronic, underlining atrophic gastritis.

The long-term prognosis of the elderly was poor when compared with the middle-aged patients in the present study. This poor prognosis for the elderly was evident not only in the overall population of patients, but in the patients who underwent a curative resection only. In addition, patients who died of causes other than gastric cancer were excluded from our prognostic analysis. Thus, we can conclude that the results obtained in this study were appropriate for evaluating prognostic differences between elderly and middle-aged patients. Similar results have been reported previously by other authors (Oohara *et al.*, 1984; Bittner *et al.*, 1985). In general, the poor prognosis can be attributed to delays in the diagnosis and the aggressiveness of the tumour. However, our study cannot address those considerations because there was no difference in the tumour staging and the histological aggressiveness of the tumour between the elderly and the middle-aged groups. One possible explanation for the poor prognosis in the elderly is a weakened host-defence status. It is likely that, as patients advance in age, they have reduced tolerance to various kinds of stress, or sometimes to cancer growth (Schwab *et al.*, 1989). With respect to post-perative death within 30 days after surgery, the elderly showed a higher death rate than middle-aged patients. Post-operative infections, which greatly affect post-operative death in elderly patients, are a result of the reduced host defence mechanism. Another explanation for the poor prognosis in the elderly is a therapeutic bias: we are more reluctant to perform aggressive treatments such as extented resection, extensive lymph node dissection and strong chemotherapy in the elderly because they cannot tolerate those treatments as well as younger patients. Indeed, for elderly patients we have consciously refrained from extensive lymph node dissection and the chemotherapy that has been conventionally performed for patients under 70 years of age (Hagiwara *et al.*, 1992). Although recent advances in perioperative management have contributed greatly to an improved prognosis for the elderly to some degree, we must now realise that this is not sufficient. To further improve the prognosis for elderly patients with gastric cancer, advances in perioperative management and more careful surgical techniques will be required.

### References

BITTNER R, SCHIRROW H, BUTTNERS M, ROSHER R, KRAUTZBERGER W, OETTINGER W AND BERGER HG. (1985). Total gastrectomy – a 15-year experience with particular reference to the patient over 70 years of age. *Arch. Surg.*, **120,** 1220 – 1125.

BLOSS RS, MILLER TA AND COPELAND EM. (1980). Carcinoma of the stomach in the young adult. *Surg. Gynecol. Obstet.*, **150,** 883 – 886.

GEHAN EA. (1965). A generalized Wilcoxon test for comparing arbitrarity single-censored samples. *Biometrika*, **52,** 203 – 224.

GRABIEC J AND OWEN DA. (1985). Carcinoma of the stomach in young persons. *Cancer*, **56,** 388 – 396.

HAGIWARA A, TAKAHASHI T, KOJIMA O, SAWAI K, YAMAGUCHI T, YAMANE T, TANIGUCHI H, KITAMURA K, NOGUCHI A, SEIKI K AND SAKAKURA C. (1992). Prophylaxis with carbon-absorbed mitomycin against peritoneal recurrence of gastric cancer. *Lancet*, **339,** 629 – 631.

HANAI A, AJIKI W, TSUKUMA H, HIYAMA T AND FUJIMOTO I. (1994). Epidemiology of gastric cancer in Osaka, Japan. Trends of incidence by histology and survival improvement. In *First International Gastric Cancer Congress*, Nishi M, Sugano H and Takahashi T. (eds.) pp. 35 – 39. Monduzzi Editore: Bologna.

HEALTH AND WELFARE STATISTICS ASSOCIATION. (1994). Death rates of malignant neoplasm by site for each sex, 1960 – 1992. In *Health and Welfare in Japan*. pp. 70 – 71. Health and Welfare Association: Tokyo.

IHAMAKI T, SANKKONEN M AND SIURALA M. (1978). Long-term observation of subjects with normal mucosa and superficial gastritis: results of 23 – 27 years' follow-up examinations. *Scand. J. Gastroenterol.*, **13,** 771 – 775.

JAPANESE RESEARCH SOCIETY FOR GASTRIC CANCER. (1981*a*). The general rules for the gastric cancer study in surgery and pathology. Part 1. *Jpn. J. Surg.*, **11,** 127 – 139.

JAPANESE RESEARCH SOCIETY FOR GASTRIC CANCER. (1981*b*). The general rules for the gastric cancer study in surgery and pathology. Part 2. *Jpn. J. Surg.*, **11,** 140 – 145.

KITAOKA H, SUEMASU K AND HIROTA T. (1972). Adhesive forces between cells and liver metastasis in cases of gastric cancer. *Jpn. J. Cancer Clin.*, **18,** 534 – 537.

LAUREN P. (1965). The two histological main types of gastric carcinoma: diffuse and so-called intestinal type carcinoma. *Acta Pathol. Microbiol. Immunol. Scand.*, **64,** 31 – 49.

MATSUSAKA T, SOEJIMA K, KODAMA Y, SAITO T AND INOKUCHI K. (1976). Carcinomas of the stomach in the young adults. *Jpn. J. Surg.*, **6,** 170 – 177.

MINISTRY OF HEALTH AND WELFARE. (1992). General mortality. In *Vital Statistics of Japan 1992*. pp. 278 – 279. Ministry of Health and Welfare in Japan: Tokyo.

MOERTEL CG, BARGAIN JA AND SOULE EH. (1957). Multiple gastric cancers. *Gastroenterology*, **32,** 1095 – 1103.

MORSON BC. (1955). Carcinoma arising from areas of intestinal metaplasia in the gastric mucosa. *Br. J. Cancer*, **9,** 377 – 385.

NOGUCHI Y, OHTA H, TAKAGI K, IKE H, TAKAHASHI T, OHASHI I, KUNO K, KAJITANI T AND KATO Y. (1985). Synchronous multiple early gastric carcinomas: a study of 178 cases. *World J. Surg.*, **11,** 127 – 139.

OOHARA T, JOHJIMA Y, YAMAMOTO O, TOHMA H AND KONDO Y. (1984). Gastric cancer in patients above 70 years of age. *World J. Surg.*, **8,** 315 – 320.

SCHWAB R, WALTERS CA AND WEKSLER ME. (1989). Host defence mechanisms and aging. *Semin. Oncol.*, **16,** 20 – 77.

TSO PL, BRINGAZE WL, DAUTERIVE AH, CORREA P AND COHN I. (1987). Gastric carcinoma in the young. *Cancer*, **59,** 1362 – 1365.